# Schools for the Sons of Medical Men

**Published:** 1845-04

**Authors:** 


### INTELLIGENCE.

### SCHOOLS FOR THE SONS OF MEDICAL MEN.

We are happy to say that Mr. Martin's admirable scheme for ensuring to the sons and near relatives of medical men the advantages of a first-rate education, at a comparatively small cost, goes on prosperously, notwithstanding the pre-occupation of the professional mind, at this time, by the many absorbing topics relating to medical polity. The particulars of this scheme are well known to all our readers. We will only state, in explanation of the terms employed in the following List of Contributions to the Schools, that the Shares are of the amount of £25 each; that Shareholders and Donors have nearly equal privileges, only that the latter incur none of the pecuniary liabilities attaching to the holders of shares in joint-stock companies; and that Benefactors neither obtain privileges (except the privilege of doing good) nor incur responsibilities.

| Shareholders. | | Numbers of Shares. |
|---|---|---|
| Edward Daniell | Newport Pagnel | 1 |
| Thomas Martin | Reigate | 1 |
| Edward Wallace | Carshalton | 1 |
| Thomas Nunneley | Leeds | 1 |
| Francis William Pittock | Sellinge, Ashford | 1 |
| John Ch. W. Lever, M.D. | Wellington street, Southwark | 1 |
| George Rogers | Bristol | 2 |
| William Alexander Greenhill, M.D. | Oxford | 1 |
| William Henchman Crowfoot | Beccles | 1 |
| R. B. Todd, M.D. | Parliament street | 1 |
| Edward Westall | Croydon | 1 |
| Henry Pout | Yalding | 2 |
| William Saubrey | Leeds, Maidstone | 1 |

| Donors. | | Amount of Donations. |
|---|---|---|
| Archibald Robertson, M.D. | Northampton | £100 |
| Thomas Martin | Reigate | 100 |
| Joseph Hodgson | Birmingham | 100 |
| Sir Benjamin Brodie, Bart. | London | 100 |
| John Forbes, M.D. | London | 100 |
| Alfred Hardwick, M.D. | Kensington | 20 |
| Sir James Clark, Bart. | London | 100 |
| Peter John Martin | Pulborough | 25 |
| William James West | Tunbridge | 20 |
| Edward Westall | Croydon | 100 |
| Sir Charles Mansfield Clarke, Bart. | London | 100 |
| Thomas Smith | Crawley | 25 |
| Edward Scholfield, M.D. | Doncaster | 20 |
| Henry Jephson, M.D. | Leamington | 105 |

| Benefactors. | | Amount of Benefactions. |
|---|---|---|
| The Misses Wallace | Carshalton | £25 |
| Edward Wallace | Carshalton | 20 |
| William Newnham | Farnham | 25 |
| The Venerable Archdeacon Pott | Exeter | 10 |

